# From microRNA target validation to therapy: lessons learned from studies on BDNF

**DOI:** 10.1007/s00018-015-1836-z

**Published:** 2015-01-20

**Authors:** Kärt Varendi, Kert Mätlik, Jaan-Olle Andressoo

**Affiliations:** Institute of Biotechnology, University of Helsinki, 00014 Helsinki, Finland

**Keywords:** microRNA, 3′UTR, RNA secondary structure, Luciferase assay, AntagomiRs, BDNF

## Abstract

During the past decade, the identification of microRNA (miR) targets has become common laboratory practice, and various strategies are now used to detect interactions between miRs and their mRNA targets. However, the current lack of a standardized identification process often leads to incomplete and/or conflicting results. Here, we review the problems most commonly encountered when verifying miR–mRNA interactions, and we propose a workflow for future studies. To illustrate the challenges faced when validating a miR target, we discuss studies in which the regulation of brain-derived neurotrophic factor by miRs was investigated, and we highlight several controversies that emerged from these studies. Finally, we discuss the therapeutic use of miR inhibitors, and we discuss several questions that should be addressed before proceeding to preclinical testing.

## Introduction

**Fig. 1:**
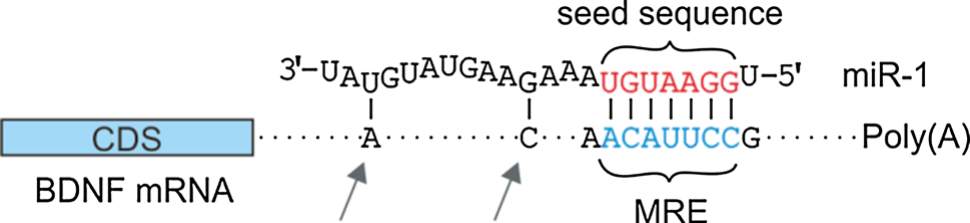
Schematic of a miR binding to its MRE within the target mRNA. The second miR-1-binding site in the *BDNF* 3′UTR is used as an example. The miR seed sequence (the nucleotides at positions 2 through 8) is shown in *red,* and the miR-binding site (MRE) in the 3′UTR is shown in *blue*. The *arrows* depict 3′ supplementary base-pairing. *MRE* miR responsive element, *BDNF* brain-derived neurotrophic factor, *CDS* coding DNA sequence

MicroRNAs (miRs) are short noncoding RNA molecules that bind their target mRNA via a short (6–8 mer) seed sequence located in their 5′ end (Fig. 1); upon binding, the miR regulates the target gene’s expression by destabilizing the mRNA and/or inhibiting its translation [1–6]. The following requirements for canonical interactions between a miR and its mRNA target sequence have been established: (1) the seed sequence must be fully complementary to the miR response element (MRE) for the miR to exert its effect; (2) as a general rule, 8-mer seed sites are more effective than 7-mer or 6-mer sites [7–9], although other means of target recognition—such as base-pairing in the central region of the miR [10] or tolerance of loops in the mRNA–miR duplex [11]—have been described; (3) 3′ complementarity between the miR and its mRNA target can facilitate repression of the target mRNA [11]; (4) in general, evolutionarily conserved MREs are regulated more effectively by miRs [7]; and (5) local sequence context—in particular, the density of adenine (A) and uracil (U) nucleotides—influences the functionality of the predicted miR-binding site [9].

These properties enable researchers to generate computational algorithms that can be used to predict the interaction between a miR and its potential target(s). Importantly, however, some target sites with a high likelihood of being regulated by miRs (i.e., evolutionarily conserved 8-mer MRE sites) do not respond to miRs (based on luciferase reporter assays and measuring mRNA and protein levels) [9, 12]. Furthermore, the current level of knowledge does not enable researchers to incorporate the mRNA secondary structure or three-dimensional conformation into the target prediction process, nor can researchers take into account potential interactions with RNA-binding proteins that may render a predicted site inaccessible to the miR [9, 13]. An evaluation of various in silico methods for predicting miR targets has revealed that even algorithms with high specificity fail to accurately predict more than 50 % of targets (reviewed in [14]), underscoring the need to experimentally verify each predicted interaction.

Here, we will first discuss briefly the methods used to verify miR targets, and some of the aspects of the experimental setup that may influence the outcome and/or reproducibility of the experiments. Next, we illustrate the above-mentioned factors by reviewing published studies regarding brain-derived neurotrophic factor (BDNF) and miR interactions, and we propose a workflow for future studies aimed at improving the strength and reliability of the results. Finally, we highlight several open questions related to translating current knowledge to preclinical testing.

## Materials and methods

The superior cervical ganglia were dissected from P1 NMRI mice, dissociated, and cultured for 14 days on a laminin-coated dish in Neurobasal medium supplemented with 2 % B-27, 0.5 mM  l-glutamine, 0.2 % Primocin, and 30 ng/ml mouse nerve growth factor (#G5141; Promega). Immediately prior to microinjection, the medium was changed to Leibovitz’s-L15 medium (#11415-06,4; Life Sciences) supplemented with 30 ng/ml mouse nerve growth factor. The cells were microinjected with the following antagomiR oligos: a 21-mer phosphodiester oligonucleotide containing a 3′-FAM (carboxyfluorescein) label (#199005-08; Exicon) or a 21-mer phosphorothioate oligonucleotide containing a 5′-FAM label (#199002-04; Exicon); both oligonucleotides contained several LNA bonds. The antagomiRs were designed with a sequence that is not complementary to any know miRs in human or mouse cells. The antagomiRs were diluted to 10 µM in phosphate-buffered saline containing 2 mg/ml 70-kDa dextran conjugated to Texas Red (#D1830, Molecular Probes) and injected into the cytoplasm of the neurons. Images were taken immediately after injection and at the indicated time points, and the images shown in Fig. 4 are representative of six successfully injected neurons for each antagomiR.

### Screening miR–mRNA interactions using the luciferase reporter assay

Reporter assays are commonly used as the first step in validating the interaction between miRs and target mRNAs; these assays are usually based on a luciferase-encoding gene derived from the sea pansy (*Renilla reniformis*) or a Photinini firefly (e.g., *Photinus pyralis*). In most cases, the 3′UTR fragment containing the MRE of interest is cloned downstream of a luciferase gene, and this reporter construct is then co-transfected into a cell line together with the pre-miR. Target recognition is presumed to have occurred if the miR in question affects the luciferase signal differently than a control miR that lacks a predicted binding site within the reporter. However, often a fragment of the 3′UTR fragment—rather than the entire 3′UTR—is used to identify candidate miR targets, which can lead to false-positive or false-negative results. The secondary structure of a short 3′UTR fragment likely differs from the structure of the full-length 3′UTR, particularly if the full-length 3′UTR is long, which is the case with the majority of genes predicted to be regulated by miRs [15]. Thus, a site that would normally be inaccessible to miRs may become available if a short fragment of the 3′UTR is used, yielding false-positive “hits”; alternatively, an accessible site may become inaccessible, yielding a false-negative result [9]. In addition, the structure of the reporter gene’s mRNA can influence the structure of the 3′UTR being studied, advocating the use of several reporters (for example, luciferase genes obtained from both *Renilla reniformis* and *Photinus pyralis*) in the assay. Finally, it is important to note that transient transfection usually yields high intracellular concentrations of the complementary molecules, which can drive interactions that would not occur under physiological conditions [16]. Therefore, additional strategies such as validating the direct interaction, overexpressing the miR, and/or suppressing the expression of endogenous miR should be used to be able to conclude that a given mRNA is indeed a target of the miR under study.

### Analysis of endogenous miR–target interactions

miRs can be overexpressed using miR precursors (pre-miRs), viral miR or pre-miR expression, plasmids encoding pre-miRs, or cell lines that stably overexpress miRs. Ideally, such studies should include verification that the introduced miR is expressed at increased levels. If the target transcript is indeed regulated by the miR in question, overexpression of the miR should decrease the target gene expression.

The expression and/or effect of a miR can be suppressed using chemically modified antisense nucleotides [17–19], and other approaches—such as miR sponges [20], target site protectors [21, 22], and miR “touch decoys” [23, 24]—are also available. If the target is regulated by an endogenous miR, and if this miR is expressed in the cells, inhibiting the miR should increase the target gene expression levels. Based on the above-mentioned factors, these complementary approaches are necessary in order to confirm that miRs identified using the luciferase assay regulate their target genes under physiological conditions. Importantly, because the mRNA and protein levels of individual genes are not necessarily correlated, both levels should be measured when a miR is overexpressed or suppressed.

### Selecting the model system to analyze endogenous miR–target interactions

Selecting a cell-based model to assess the effects of miRs on their targets is not trivial. Because primary cell cultures are more likely to recapitulate in vivo conditions, they are clearly superior to cell lines in terms of assessing miR–target interactions in a physiological context. However, preparing primary cultures can be both time-consuming and labor-intensive, and difficulties delivering the miR—coupled with potentially low expression of the putative targets—may complicate the analysis of miR–target interactions. For these reasons, immortalized cell lines are often used in in vitro studies. Nevertheless, it is important to bear in mind that cell lines derived from cancerous tissues can have aberrant levels of gene expression, which will likely affect miR-mediated target regulation. For example, the results could be affected by the presence or absence of RNA-based or protein-based cofactors necessary for miR binding, or by the presence of RNA-binding proteins that modulate site accessibility. Furthermore, many of the genes that are subject to miR-mediated suppression are regulatory in nature [15], and their temporally and/or spatially complex expression pattern is not necessarily recapitulated in a cell line grown under fixed culture conditions. Thus, when previous knowledge regarding the function of the target gene is known, the preferred approach is to choose a cell line that is likely to retain at least some of the original tissue’s “normal” gene expression profile. Moreover, several cell lines can be used, thereby minimizing the likelihood of identifying false-positive and/or false-negative interactions.

In addition to choosing the optimal cell culture system, the experimental timeframe is also likely to influence the outcome of miR transfection. For example, it can take 8–10 h to maximally load Argonaute proteins (a family of proteins that direct miR–target binding and subsequently block translation and/or mRNA cleavage) with small RNAs [25]. Furthermore, the median half-life of mRNAs and proteins is 9 and 46 h, respectively [26]; therefore, mild changes in gene expression following miR transfection may not be detectable if the change is measured too early after transfection (e.g., under 24 or even 48 h), particularly when measuring protein levels.

### Validation of the direct interaction

Although performing miR overexpression and miR inhibition experiments are important steps in verifying miRs as regulators of candidate genes, these experiments do not reveal whether an observed change in target gene levels is the result of direct binding between the miR and the predicted site in the 3′UTR. This can be analyzed in a luciferase assay by co-expressing the miR and the 3′UTR containing a mutated MRE site. Unfortunately, however, direct miR–mRNA interactions are tested only rarely. To illustrate this point, Table 1 summarizes the data collected to date from *BDNF* mRNA–miR studies. Despite the presence of a putative conserved binding site within a given gene, the responsive 3′UTR might still be regulated indirectly by other targets of the miR. Furthermore, several studies have used a strategy in which miR seed sites are mutated and the effect on a reporter or endogenous gene is compared to the effect of the wild-type miR. Although demonstrating that the mutated miR has no effect on the target gene indicates that the target gene is regulated by the miR in question, such an experiment does not necessarily confirm that the miR interacts directly with the given 3′UTR. In this respect, the results are no more informative than results obtained from either exogenous miR overexpression or miR suppression. Therefore, the predicted MRE site in the 3′UTR should be mutated in the reporter construct in order to determine whether the effect of the miR is direct or indirect.

**Table 1:** Current knowledge regarding the regulation of the 3′UTR of the *BDNF* mRNA by miRs based on studies that used a luciferase reporter assay

MicroRNA	3′UTR sequence used	Effect on luciferase signal in the reporter assay	Direct interaction shown	References
miR-1	Long and short 3′UTR	↓^b^	Yes (sites #1 and #2 are functional)	[27]
	Synthetic oligo^a^	↓^b^	Yes^h^	[77]
miR-10b	Long and short 3′UTR	↓^b^	Yes	[27]
miR-15a	Long and short 3′UTR	No effect^b^	n.d.	[27]
	855-nt 3′UTR fragment	↓^c^	n.d.	[94]
miR-16	Long and short 3′UTR	No effect^b^	n.d.	[27]
miR-22	Long 3′UTR	↓^d^	n.d	[95]
miR-26a, 26b	Long 3′UTR, including ca 30 nt of the CDS	↓^d^	Yes	[96]
miR-30a	Long and short 3′UTR	No effect^b^	n.d	[27]
	552-nt 3′UTR fragment	↓^b^	n.d	[97]
miR-30b	Long and short 3′UTR	No effect^b^	n.d	[27]
	552-nt 3′UTR fragment	No effect^b^	n.d	[97]
miR-30c	552-nt 3′UTR fragment	No effect^b^	n.d	[97]
miR-107	552-nt 3′UTR fragment	No effect^b^	n.d	[97]
miR-138-2	Long 3′UTR	No effect^d^	n.d	[95]
miR-155	Long and short 3′UTR	↓ (long 3′UTR only)^b^	yes	[27]
miR-182	Long and short 3′UTR	No effect^b^	n.d	[27]
miR-191	Long and short 3′UTR	↓ (long 3′UTR only)^b^	yes	[27]
	469-nt 3′UTR fragment	↑^e^	n.d	[84]
	552-nt 3′UTR fragment	No effect^b^	n.d	[97]
miR-195	Long and short 3′UTR	No effect^b^	n.d	[27]
	Not specified	No effect^f^	n.d	[82]
	552-nt 3′UTR fragment	↓^b^	n.d	[97]
miR-204	Long 3′UTR	↓^b^	Yes	[92]
miR-206	Long 3′UTR	↓^c^	Yes (sites #1, #2 and #3 are functional)	[83]
	Not specified	↓^f^	n.d	[82]
	Long 3′UTR (human), 3′UTR fragments (mouse)	↓ (long 3′UTR and fragment containing site #3)^d^	Yes (only site #3 is functional)	[81]
	Short 3′UTR, 478 nt and 1,355 nt 3′UTR fragments	No effect (short 3′UTR), ↓ (long 3′UTR fragment)^g^	Yes (sites #1 and #2 are functional)	[80]
	1,500-nt 3′UTR fragment	↓^c^	n.d	[79]
	1,057-nt 3′UTR fragment	No effect^g^	n.d	[78]
miR-210	60-nt 3′UTR fragment	↓^b^	Yes	[93]
miR-339	Long 3′UTR	No effect^d^	n.d	[95]
miR-376b-5p	3′UTR fragment, size not specified	↓^c^	n.d	[100]
miR-497	Not specified	No effect^f^	n.d	[82]

*n.d* not determined

^a^Synthetic oligo with no similarity to *BDNF* mRNA containing three sites complementary to miR-1 binding site

^b^HEK-293 cells

^c^HEK-293T cells

^d^HeLa cells

^e^MCF7 cells

^f^SH-SY5Y cells

^g^C2C12 cells

^h^Shown using miR-1-binding site mutations in a synthetic oligonucleotide with no similarity to *BDNF* mRNA

A direct interaction between an endogenous miR and its target can also be confirmed using target site protectors that are designed to specifically prevent the miR from binding to its predicted target site in the 3′UTR [21, 22]. Although target site protectors have been described to prevent binding of the miR to its target in vivo [21], they are currently not widely used. We attempted to study the regulation of endogenous BDNF by endogenous miRs using morpholino antisense oligos as target site protectors; these oligos were designed to prevent the binding of miR-1 and miR-10b to the 3′UTR of the *BDNF* mRNA. We previously identified binding sites for miR-1 and miR-10b in the 3′UTR of *BDNF* and found that these sites act as direct regulators of *BDNF* via its 3′UTR [27]. However, target site protectors that mask the same sites had no effect on endogenous *BDNF* mRNA or BDNF protein levels. Thus, due to steric and/or other factors, morpholino oligos may not be effective at inhibiting all potential miR–target interactions. Despite the clear advantage of enabling gene-specific de-repression, the relative paucity of published studies that use target site protectors suggests that this method needs further development.

In addition, immunoprecipitation methods can be used to identify the target mRNAs of endogenous miRs [28]. For example, a genome-wide screen using Argonaute immunoprecipitation followed by high-throughput RNA sequencing identified thousands of putative endogenous miR–target interactions in the mouse brain [29] and in HEK-293 cells [30]. Although these results require validation using other methods, Argonaute immunoprecipitation followed by target mRNA detection methods (e.g., qPCR-based detection of the predicted target mRNA levels following pre-miR overexpression) might be a valuable tool for use in future studies. Furthermore, microarray and RNA-seq analyses of gene expression in cell lines following miR transfection/knockdown have provided additional information regarding potential miR–target interactions [3, 12, 31–34]. Together, the above-mentioned high-throughput datasets, which are currently available at http://servers.binf.ku.dk/antar/ [34], may serve as a valuable starting point for future studies.

### Measuring the concentration of endogenous miRs and their targets

The absolute levels of endogenous miRs and their predicted target transcripts can play a significant role in the degree of the target genes’ downregulation by miRs. For example, miRs that are expressed at low levels are generally ineffective at suppressing their predicted targets [35]. Therefore, the physiological relevance of verified miR–target interactions should be assessed by analyzing the ratio of the miR to its target mRNA in specific tissues and/or cell types.

On the other hand, a recent study found that the ability of a miR to suppress the expression of individual targets is not necessarily correlated to its expression level [36]. Indeed, the total number of available target transcripts can affect the potential of a miR to reduce the level of its targets. Specifically, miRs that have a high number of available target transcripts suppress each individual target to a lesser extent than miRs that have a smaller number of available targets [37]. Therefore, knowing the absolute levels of a given miR and its target mRNA may not be sufficient without knowing the expression of other transcripts that the miR might target. Thus, the absolute expression levels of the miR and its putative target in the analyzed cell type or tissue do not necessarily confirm or preclude the possibility that their interaction is physiologically significant in a specific context, and this should be investigated experimentally. Nevertheless, the miR and mRNA expression levels can be used to estimate the likelihood of such interactions.

Endogenous mRNA transcripts can be quantified relatively easily using real-time RT-PCR [38]. On the other hand, measuring the number of endogenous miR copies appears to be more challenging, given their relatively short length. RT-PCR-based miR quantification [39–41] is both cost-effective and suitable for analyzing a small number of miRs simultaneously; however, it is not efficient enough for use on a genomic scale. In contrast, high-throughput methods such as microarray hybridization [42] and next-generation sequencing [43] allow researchers to analyze hundreds of miRs simultaneously, but these methodologies tend to be relatively expensive. It is also important to note that the values of the measured miR levels can vary considerably depending on the technology used, which suggests that miR levels should be quantified using several approaches in order to increase reliability of the results [44]. Finally, databases containing information regarding miR expression levels in various tissues and cell types are continuously being expanded, and these valuable resources can be used to estimate the magnitude of a given miR expression in a given site.

### Assessing the cooperative effects of miRs

Finally, problems related to identifying and validating the target can arise because miRs often exert only a mild effect on the expression of their targets [3, 12, 45]. In other words, small changes in gene expression (i.e., on the order of 5–10 %) can be difficult to characterize as significant and/or functionally important. In addition, assessing the effect of a combination of miRs—each of which may exert only a small effect on the expression of a specific gene—can be challenging. Importantly, studies have shown that several miR sites within a single 3′UTR can repress gene expression synergistically [9, 46, 47]. To investigate the possible synergistic effect of multiple endogenous miRs on the regulation of a given target, the seed sequences of the validated miR-binding sites can be mutated alone or in combination, and this can be followed by a luciferase assay without the addition of exogenous miRs. This approach was used successfully to demonstrate that four miR-binding sites in the 3′UTR of *BDNF* are used synergistically by endogenous miRs to regulate the expression of BDNF [27]. Because miRs often act in concert to regulate individual targets [48], an analysis of the cooperative effect of miRs (for example, by replacing the gene’s 3′UTR with a 3′UTR that lacks predicted miR binding sites or by mutating most of the validated miR sites) is needed in order to obtain a more thorough understanding of how the gene expression is regulated by miRs, particularly in a physiological context.

In summary, several complementary approaches can be used to verify miR–target interactions, and these approaches can support the finding that a given mRNA is regulated by one or more specific miRs. Below, we illustrate and expand upon the above-mentioned points by summarizing and discussing the existing knowledge regarding the interaction between the *BDNF* mRNA and miRs.

## Lessons from studies on BDNF

Brain-derived neurotrophic factor (BDNF) is a target-derived neurotrophic factor that promotes the survival of several types of central and peripheral neurons [49–51]. BDNF plays a key role in the development and function of the nervous system, including synaptic plasticity, learning, and memory. Although knocking down the expression of BDNF in vitro has relatively few consequences, even a mild change in BDNF levels can have severe consequences in vivo. For example, heterozygous BDNF-knockout mice have deficits in striatal dopamine output [52], long-term potentiation [53, 54], hippocampal learning [55], and presynaptic GABAergic function [56]. Changes in BDNF levels have also been implicated in a variety of neuropsychiatric disorders, including Alzheimer’s disease [57, 58], bipolar disorder [59–61], schizophrenia [62–66], and depression [67–69]. Outside of the brain, increased BDNF expression is believed to contribute to several processes, including the generation and maintenance of neuropathic pain [70, 71] and muscle regeneration following injury [72].

### miR-mediated regulation of BDNF expression

**Fig. 2:**
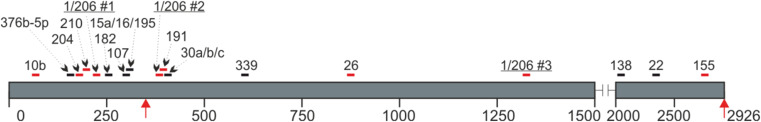
Schematic of miR-binding sites in the 3′UTR of the *BDNF* mRNA. The miR-binding sites shown in *red* have been validated as direct regulators of BDNF expression; the sites shown in *black* have been experimentally demonstrated as possible BDNF regulators, but have not been validated. The *red arrows* indicate alternative polyadenylation sites. The three predicted binding sites for miR-1/206 (1/206 #1, 1/206 #2, and 1/206 #3) are indicated. Note that the miR-376b-5p binding site is present in the rat and mouse 3′UTR, but not in the human 3′UTR

**Fig. 3:**
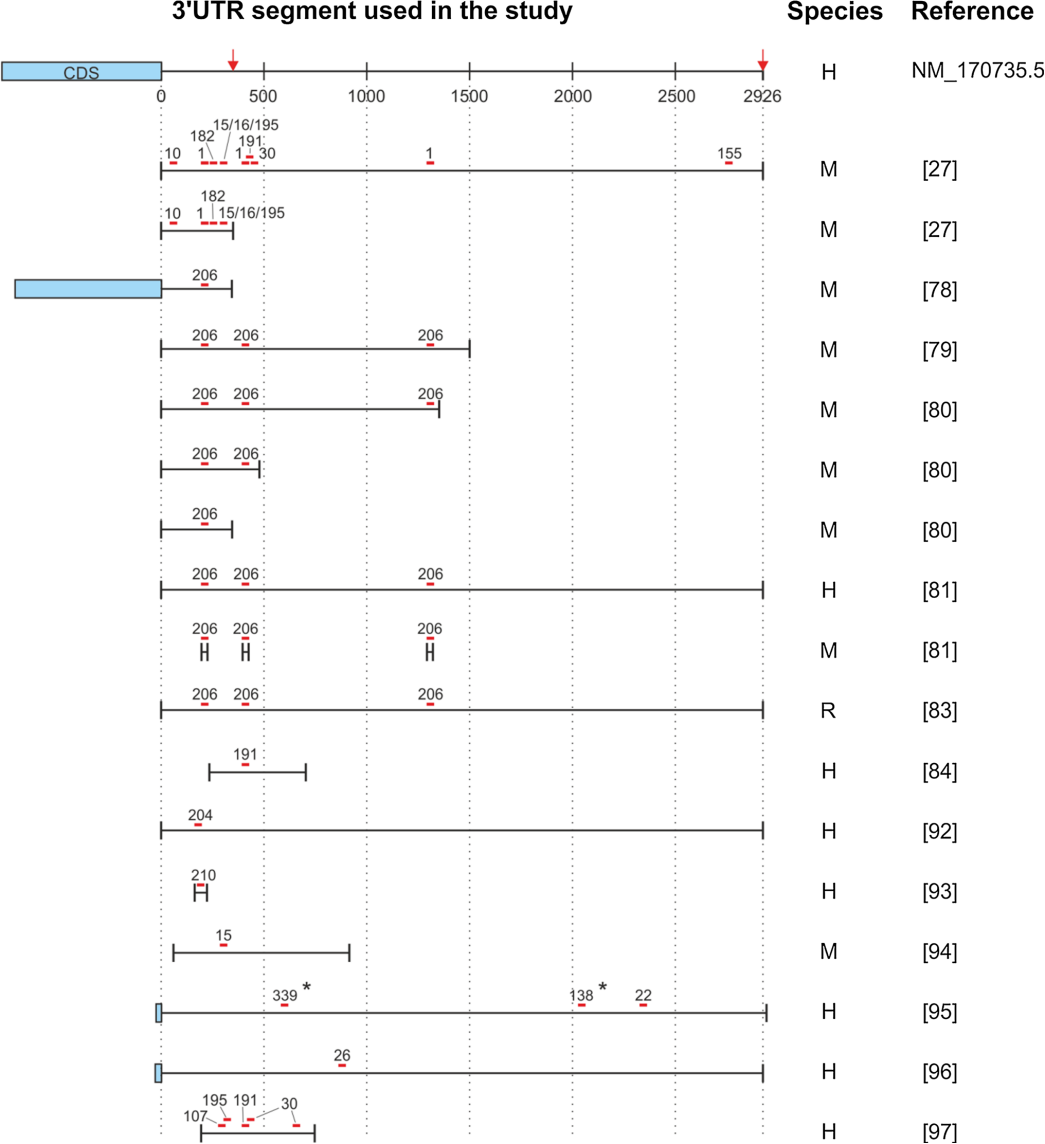
Schematic showing the 3′UTR fragments of the *BDNF* mRNA that were used to study BDNF–miR interactions. Only studies in which the size of the 3′UTR fragment was reported are included. The binding sites for the miRs examined in each study are shown in *red*. The alternative transcription stops and polyadenylation sites that yield two distinct *BDNF* mRNA isoforms (i.e., containing a short and long 3′UTR) are indicated with *red arrows*. NM_170735.5 refers to human *BDNF* mRNA, transcript variant 1, NCBI reference sequence. *H* human 3′UTR, *M* mouse 3′UTR, *R* rat 3′UTR, *Asterisk* miR-binding site present only in humans

The *BDNF* mRNA contains two alternative polyadenylated transcription stop sites, yielding two pools of transcripts that differ with respect to the length of the 3′UTR; the long *BDNF* transcript contains a ~3,000-nt 3′UTR, whereas the short *BDNF* transcript contains a ~350-nt 3′UTR (Figs. 2, 3) [73]. Interestingly, several predicted miR-binding sites are located exclusively in the long 3′UTR, providing a possible mechanism for miRs to differentially regulate the two mRNA isoforms (Fig. 2).

According to various target prediction programs, *BDNF* is potentially regulated by several hundred miRs via its 3′UTR. To date, however, only approximately 25 miRs have been investigated experimentally, each to a different extent (Fig. 2). Of the 25 potential *BDNF* mRNA–miR interactions studied, approximately one-third have been analyzed by mutating the MRE and have been confirmed as direct interactions (the red lines depicted in Fig. 2). Below, we discuss our current knowledge regarding the regulation of BDNF expression by miRs and the currently unresolved issues.

#### miR-1

miR-1 is an evolutionarily conserved member of the miR-1/206 family and is expressed specifically in muscle tissue [74–76]. Based on sequence prediction, the 3′UTR of the *BDNF* mRNA contains three putative binding sites for miR-1; one binding site is located in the short 3′UTR, and two sites are located in the long 3′UTR (Fig. 2).

Using the full-length long and short *BDNF* 3′UTR isoforms in a luciferase expression assay, we recently found that miR-1 can inhibit luciferase expression via both isoforms. Mutation analysis revealed that the first two predicted binding sites are used by miR-1; in contrast, mutating the third site did not affect the luciferase signal compared to the wild-type 3′UTR [27]. Given that miR-binding sites located near the center of the 3′UTR are generally less effective than sites located at either end of the 3′UTR [9], we hypothesize that the third site, which is located at the center of the *BDNF* 3′UTR, may not be accessible to miR-1 due to the mRNA secondary structure.

We also measured endogenous BDNF expression in human artificial retinal pigment epithelial (ARPE-19) cells, which produce high levels of BDNF. Following treatment with pre-miR-1, both the intracellular and secreted BDNF protein levels were reduced. Interestingly, we observed no change in *BDNF* mRNA levels, suggesting that miR-1 may suppress BDNF production at the level of protein synthesis [27].

The effect of miR-1 on BDNF expression was also examined in another recent study, which found that overexpressing miR-1 in U-87 MG cells (a human primary glioblastoma line) caused a 50 % decrease in BDNF protein levels [77]. The authors used a luciferase assay and transfected a 60-nt synthetic oligonucleotide containing three sites complementary to the miR-1 seed sequence flanked by a sequence that was not related to the *BDNF* 3′UTR. Mutating the three miR-1 sites increased the luciferase signal compared to a construct containing wild-type miR-1 sites. The authors concluded that their results support a direct interaction between miR-1 and the 3′UTR of *BDNF* [77]. However, because the reporter construct contained no adjacent sequences of the *BDNF* mRNA, not to mention the full-length 3′UTR, the observed interaction might not be specific to BDNF, but might reflect the ability of a miR to bind to and inhibit its MRE.

#### miR-206

miR-206 is in the same miR family as miR-1 and shares the same predicted binding sites in the 3′UTR of the *BDNF* mRNA (Fig. 2). Outside of the seed region, miR-1 and miR-206 differ by only four nucleotides. Several studies have used luciferase reporter assay to investigate the ability of miR-206 to regulate *BDNF* mRNA via its 3′UTR [78–83]. However, the results obtained to date have been contradictory, and this is likely due to differences in experimental design (see Fig. 3; Table 1). For example, using a luciferase construct containing a 1,057-nt fragment with part of the BDNF coding sequence and only the most proximal miR-206 binding site in the 3′UTR, Kim et al. [78] concluded that miR-206 does not suppress BDNF expression via its 3′UTR, although endogenous *BDNF* mRNA levels were reduced after treating C2C12 cells with miR-206. In contrast, Radzikinas et al. [79] found that a 1,500-nt *BDNF* mRNA 3′UTR fragment (containing all three predicted miR-206 binding sites) was suppressed by miR-206. Moreover, Miura et al. [80] found that the short 3′UTR of *BDNF* was not regulated by miR-206, whereas a luciferase construct containing 1,355-nt 3′UTR fragment (including all three binding sites) was suppressed by miR-206. In the same study, mutation analysis revealed that mutating each site independently had no effect on the miR-206–induced suppression of the *BDNF* 3′UTR, whereas mutating the first two sites together prevented miR-206–induced inhibition. Interestingly, Lee et al. [81] reached the opposite conclusion based on their finding that the full-length 3′UTR was inhibited by miR-206, whereas an analysis of the three binding sites using short mutant 3′UTR fragments revealed that only the third site was functional. Finally, a study by Tapocik et al. [83] provided evidence that miR-206 mediates suppression via all three putative binding sites in the full-length 3′UTR.

In summary, a clear consensus is currently lacking regarding the effect of miR-206 on the long 3′UTR of the *BDNF* mRNA, and the function of each predicted binding site differs among studies. Therefore, it is highly likely that the use of different length 3′UTR fragments (rather than the full-length 3′UTR) contributes significantly to the contradictory results obtained from these studies. Thus, until we thoroughly understand the three-dimensional structure of the 3′UTR (and its effect on the miR-mRNA interaction), data obtained from fragments of the 3′UTR should not be used to draw conclusions based on the full-length 3′UTR.

Interestingly, experimental evidence suggests that the short 3′UTR of *BDNF* may not be regulated by miR-206 [78, 80], despite the presence of a putative binding site. This finding warrants further attention, as miR-1—which shares an MRE with miR-206 but differs from miR-206 by only four nucleotides outside the seed sequence—does suppress *BDNF* mRNA via the short 3′UTR [27]. Thus, future studies of the *BDNF* 3′UTR may yield further insight regarding how the sequence and secondary structure of the mRNA regulate the effects of miRs on their specific target.

#### miR-10b

We recently reported that miR-10b has a single, highly conserved 8-mer binding site in the short 3′UTR of the *BDNF* mRNA. Using a luciferase assay, we found that miR-10b suppressed reporter expression via both the long and short 3′UTRs of *BDNF*; moreover, mutating the putative binding site in the long 3′UTR abolished suppression induced by overexpressed miR-10b and endogenous miR-10b. In addition, we used ARPE-19 cells to show that endogenous *BDNF* mRNA and BDNF protein levels are: (1) decreased following transfection with pre-miR-10b and (2) increased after endogenous miR-10b was downregulated using an antagomiR that targets miR-10b. Taken together, these data suggest that miR-10b is a direct regulator of BDNF expression [27].

#### miR-155 and miR-191

Both miR-155 and miR-191 have predicted binding sites in the long 3′UTR of the *BDNF* mRNA (Fig. 2). Using a luciferase assay, we recently reported that both miR-155 and miR-191 specifically reduce the expression of a luciferase construct containing the *BDNF* long 3′UTR but not the short 3′UTR. Moreover, mutation analysis revealed that the effect on gene expression is mediated directly via the predicted MREs. We also measured BDNF levels after transfecting two neural cell lines (ARPE-19 and U-87 MG cells) with pre-miRs (Table 2). The majority of *BDNF* transcripts in these cells contain the short 3′UTR, which should not respond to miR-155 or miR-191. Consistent with this notion, we found that the expression of *BDNF* mRNA isoforms carrying the long 3′UTR was reduced following treatment with miRs-155 and miR-191 precursors, although total *BDNF* mRNA and BDNF protein levels were unaffected [27].

**Table 2:** Current knowledge regarding the regulation of endogenous BDNF levels by miRs

MicroRNA	Effect of miR overexpression on BDNF levels compared to control		Effect of miR suppression on BDNF levels compared to control		References
	BDNF mRNA	BDNF protein	BDNF mRNA	BDNF protein	
miR-1	20 % ↓ (long 3′UTR only)^a^	20–40 % ↓^a^	n.d	n.d	[27]
	No effect^b^	20–40 % ↓^b^	n.d	n.d	[27]
	n.d	50 % ↓^a^	n.d	n.d	[77]
	↓^k^	n.d	n.d	n.d	[78]
miR-10b	20 % ↓ (long 3′UTR only)^a^	No effect^a^	100 % ↑^a^	20–30 % ↑^a^	[27]
	25 % ↓^b^	15–40 % ↓^b^	n.d	n.d	[27]
miR-30a	No effect^c^	30 % ↓^c^	n.d	n.d	[97]
miR-124a	50 % ↓^d^	30-40 % ↓^d^	n.d	n.d	[98]
miR-132	n.d	25 % ↓^e^	n.d	n.d	[99]
miR-155	20–30 % ↓ (long 3′UTR only)^a b^	No effect^a b^	n.d	n.d	[27]
miR-182	n.d	0–40 % ↓^e^	n.d	n.d	[99]
miR-191	20-30 % ↓ (long 3′UTR only)^a^	No effect^a^	n.d	n.d	[27]
	No effect^b^	No effect^b^	n.d	n.d	[27]
	40 % ↑^m^	n.d	60 % ↓^m^	n.d	[84]
miR-204	70 % ↓^f^	80 % ↓^f^	150 % ↑^f^	100 % ↑^f^	[92]
miR-206	50 % ↓^g^	n.d	n.d	n.d	[110]
	n.d	50 % ↓^c^	n.d	100 % ↑^c^	[111]
	n.d	n.d	n.d	50 % ↑^c^	[83]
	n.d	↓^e^	n.d	↑^e^	[82]
	n.d	↓^e h i j^	n.d	↑^e h i j^	[81]
	50 % ↓ (long and total 3′UTR)^k^	n.d	60 % ↑^k^	n.d	[80]
	↓^k^	n.d	n.d	n.d	[78]
miR-210	n.d	80 % ↓^f^	n.d	300 % ↑^f^	[93]
miR-376b-5p	n.d	30 % ↓^l^	n.d	No effect^l n^	[100]

*n.d* not determined

^a^U-87 MG cells

^b^ARPE-19 cells

^c^Rat primary neuronal cultures

^d^NG108-15 cells

^e^SH-SY5Y cells

^f^HEK293 cells

^g^SGC-7901 (cell line stably expressing miR-206)

^h^Neuro2a cells

^i^bEnd.3 cells

^j^HUVEC (human umbilical vein endothelial cells)

^k^C2C12 cells

^l^H9c2 cells

^m^MCF7 cells

^n^Compared to control; a difference in miR suppression was observed between miR-376-5p + miR-376-5p inhibitor and miR-376-5p alone

In contrast to the above-mentioned study, Nagpal et al. [84] found that overexpressing miR-191 increased the expression of a luciferase reporter construct containing a 475-nt fragment of the *BDNF* 3′UTR, which contains the putative miR-191 site. In addition, overexpressing miR-191 increased the expression of endogenous BDNF in MCF7 cells (a breast cancer cell line), and suppressing endogenous miR-191 expression using a specific antagomiR decreased BDNF levels. However, whether the effect of miR-191 effect on BDNF expression is direct was not investigated [84]. Given that the dysregulation of miR-191 [85, 86] and BDNF [87–91] levels vary among different tumor types, regulatory cofactors may determine whether miR-191 suppresses or activates the expression of BDNF.

#### miR-204

Recently, Imam et al. thoroughly examined the role of miR-204 in regulating BDNF expression. Endogenous BDNF mRNA and protein levels were reduced after miR-204 overexpression and increased after inhibition of endogenous miR-204. Furthermore, miR-204 suppressed luciferase expression via the full-length *BDNF* 3′UTR, and mutating the predicted binding site abolished repression by miR-204, suggesting that BDNF is a direct target of miR-204 [92].

#### miR-210

Using bioinformatics, Fasanaro et al. [93] identified BDNF as a potential target of miR-210. However, in human umbilical vein endothelial cells (HUVEC), neither *BDNF* mRNA nor BDNF protein levels were changed by overexpressing miR-210 or suppressing endogenous miR-210 expression. On the other hand, using HEK-293 cells the same group found that overexpressing miR-210 and treating cells with antagomiR-210 reduced and increased BDNF protein levels, respectively. Finally, experiments with a luciferase construct containing either the wild-type or seed-deleted 60-nt 3′UTR fragment revealed that miR-210 binds directly to the predicted site in the 3′UTR of *BDNF* mRNA [93]. In summary, the evidence to date suggests that BDNF is a target of miR-210 under certain conditions.

### Other putative BDNF-regulating miRs

In addition to the aforementioned miRs, some studies have suggested that BDNF expression is regulated by other miRs as well. The following miRs have been proposed as putative regulators of BDNF: miR-15a [94], miR-22 [95], miR-26a and miR-26b [96], miR-30a [97], miR-124 [98], miR-132, miR-182 [99], miR-195 [97], and miR-376b-5p [100]. Unfortunately, these studies lack evidence regarding (1) whether the miR–mRNA interactions are direct and/or (2) the effect of the respective miR on endogenous BDNF expression (see Tables 1, 2). In addition, some of the reported *BDNF* mRNA–miR interactions have not been confirmed by independent studies [27, 82], further complicating the situation and underscoring the need for a uniform system for validating the target.

## From in vitro target validation to in vivo function

Given that reduced BDNF levels are associated with several neurological and neuropsychiatric disorders, miRs that inhibit the expression of BDNF are attractive targets for clinical studies. However, among the miRs that have been shown to regulate BDNF in vitro, only miR-206 has been reported to regulate BDNF levels in vivo [79, 81, 83].

In Alzheimer’s disease (AD), the expression of BDNF is reduced [101, 102]. In addition, BDNF has a protective effect against amyloid β1–42 toxicity in cultured neurons [103] and has beneficial effects in primate and rodent models of AD [104]. In addition, Lee et al. [81] attempted to increase BDNF levels in the brains of Tg2576 mice, a mouse model of AD. Tg2576 mice overexpress a mutant form of amyloid precursor protein; as a result, they develop amyloid β plaques and impaired hippocampal function, both of which are associated with deficits in cognitive function [105]. Using a combination of microarray analysis, real-time PCR, and in situ hybridization, Lee et al. found that the expression of miR-206 is increased in the brains of Tg2576 mice. They also used RT-PCR to show that miR-206 was upregulated in the temporal cortex in the brains of patients with AD. Because in vitro experiments suggested that BDNF is a direct target of miR-206, they investigated the function of miR-206 in vivo by injecting 0.5 nmol of Cy3-labeled 2′-*O*-methyl antagomiR-206 (AM206) into the third ventricle of 12-month-old Tg2576 mice, resulting in the widespread distribution of AM206 throughout the hippocampus and surrounding tissues after 24 h (shown using Cy3 fluorescence). One week after AM206 injection, BDNF levels were increased in the hippocampus, striatum, and cortex. AM206 injection caused improved performance in behavioral tests that assess memory. Furthermore, intranasal delivery of AM206 in Tg2576 mice also elevated BDNF levels in several brain regions—including the hippocampus, striatum, and cortex—and increased hippocampal memory function [81].

The findings from the above-mentioned study have clear therapeutic potential. Unfortunately, however, data regarding the pharmacodynamics of antagomiRs, including the tissue distribution over time, cell type specificity, stability, clearance, and toxicity in the brain, were not investigated. In various tissues, 2′-*O*-methyl oligonucleotides have been shown to reduce expression of their target miR for ≥3 weeks [106]; however, in their study, Krutzfeldt et al. [106] found that the antagomiRs effectively reduced target gene levels in all tissues tested except the brain, suggesting that intravenously injected antagomiRs do not reach the brain or are less effective in the brain. Recently, Jimenez-Mateos et al. [107] reported that an intracerebroventricular injection of locked nucleic acid (LNA)–based antagomiRs affected endogenous miR expression in the hippocampus within 12 h. By 24 h, endogenous miR expression was reduced by 95 % compared to control-treated animals, and expression was still reduced by 50 % 1 month after antagomiR injection; miR expression returned to baseline levels 2 months after treatment [107], suggesting that antagomiRs cause long-term silencing of their target miRs in the brain, similar to other tissues [106].

In their respective studies, Lee et al. [81] and Jimenez-Mateos et al. [107] did not investigate the fate of antagomiRs in the brain; to date, antagomiR processing and metabolism in the brain have not been examined. Thus, several key questions remain. How do antagomiRs get into cells in the brain? In which intracellular compartment(s) do antagomiRs reside, and for how long? How specific are their effects on target miRs? Do antagomiR levels correlate temporally with the levels of their target miR and/or the levels of the miR targets? What are the long-term consequences of antagomiR treatment on gene expression and behavior?

**Fig. 4:**
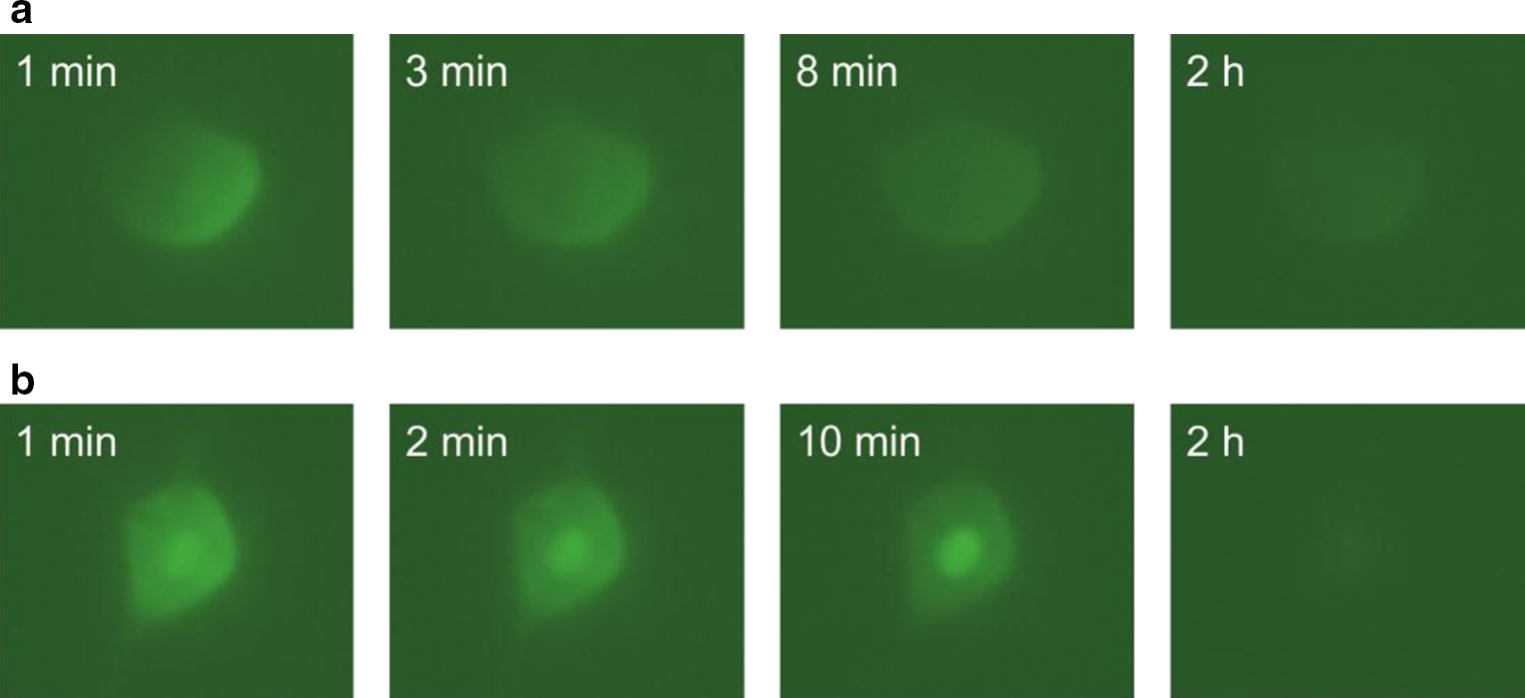
Fluorescent signal measured from FAM-labeled LNA-based antagomiRs. The labeled antagomiR was microinjected into primary superior cervical ganglion neurons isolated from neonatal (P1) mice. The images were taken at the indicated times relative to microinjection, and representative images are shown (*n* = 6 cells per experiment). **a** Oligonucleotides on a phosphorothioate backbone diffused slowly throughout the cytoplasm. Within several minutes, the signal was distributed weakly throughout the entire cell. **b** Oligonucleotides on a phosphodiester backbone diffused rapidly throughout the cytoplasm and accumulated into the nucleus. Over time, the signal became weaker in the cytoplasm but remained strong within the nucleus

Results from our laboratory suggest that the fluorescent signal from fluorophore-labeled LNA-based antagomiRs decreases rapidly (i.e., within minutes) following direct microinjection into primary sympathetic neurons (Fig. 4). We also found that oligonucleotides with phosphodiester and phosphorothioate backbones have distinct temporal patterns of intracellular localization (Fig. 4). These results raise several intriguing questions. For example, why does the signal emitted by fluorophore-labeled LNA-based oligonucleotides decrease within minutes in primary neurons cultured in vitro, whereas intracranially injected fluorescent signals can last several days (or weeks) in vivo [81, 106]? Does the antagomiR remain linked to the fluorophore both in vitro and in vivo? Do these properties influence the stability of antagomiRs and/or their effect on endogenous miRs? These are but a few of the important questions that must be addressed in future studies.

Toxicity from antagomiRs is another issue that must be investigated. In their recent study, Lee et al. [81] delivered two doses of intranasal AM206 (0.5 and 5 nmol); the lower dose did not increase BDNF levels. Although the high dose of AM206 caused no apparent adverse effects, it remains unclear whether inhibiting miR-206 function resulted in an undesirable upregulation of its other targets. Consistent with this possibility, Lee et al. [81] found that the level of synaptophysin—which is not a predicted target of miR-206—was also increased after the delivery of the higher dose of AM206, suggesting that the expression of additional genes may be affected. Given that miR-206 can act as a tumor suppressor in several cancers, including breast cancer [108], lung cancer [109] and stomach cancer [110], potential side effects due to miR-206 downregulation must be monitored closely and reported.

## Conclusions

**Fig. 5:**
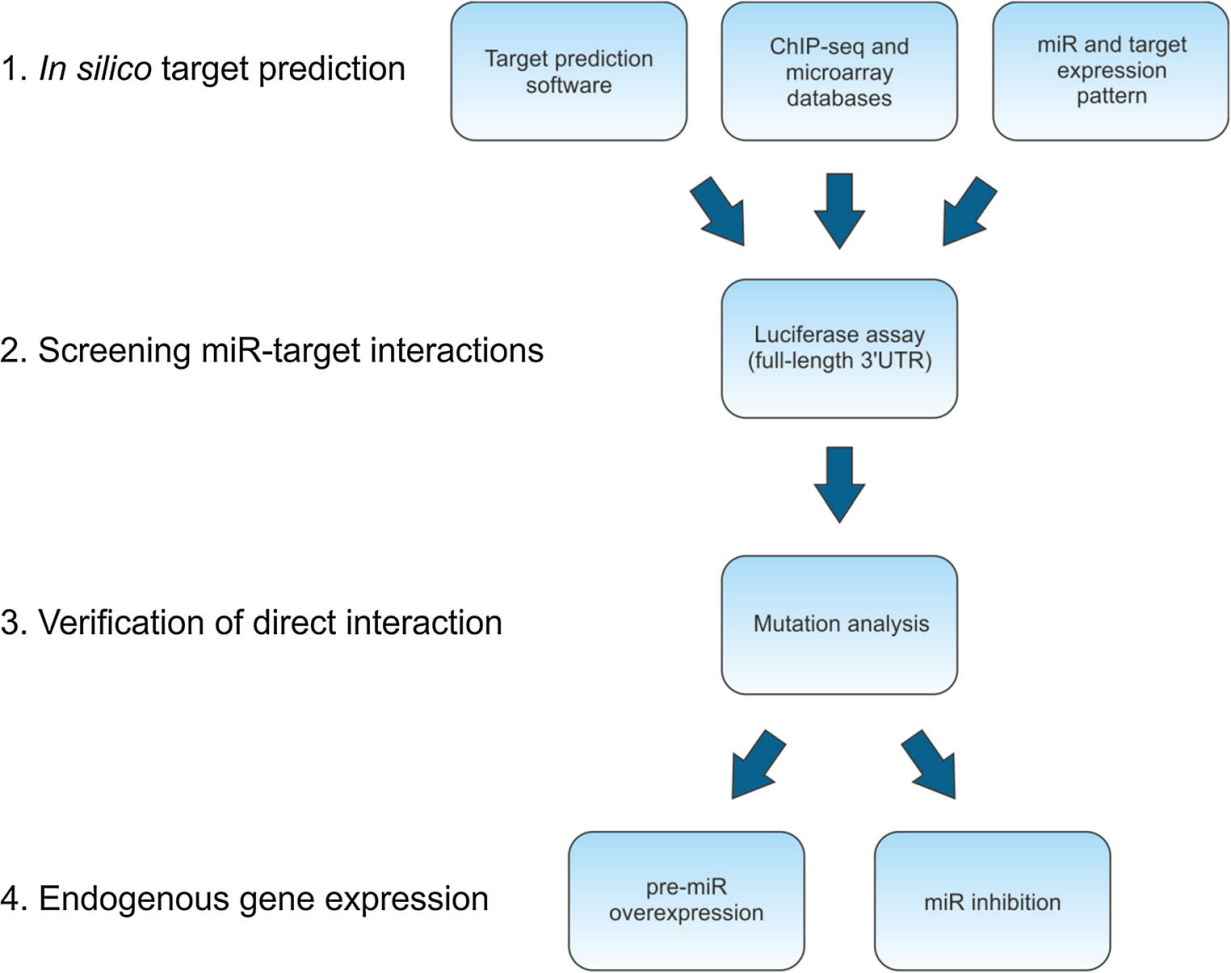
Recommended workflow plan for identifying and validating miR targets. For details, see the text

Based on in silico findings, each miR can have hundreds of putative mRNA targets. Thus, the major challenge in studying miR–target interactions is identifying which specific interactions play a functional role in vivo. Given that even minor differences in methodologies can yield contradictory results, each published miR–target interaction should be interpreted with caution, particularly when the experimental evidence is limited. Based on the issues discussed in this review, we propose a four-step standardized workflow plan for studying specific miR–mRNA interactions (Fig. 5). Moreover, we emphasize that a comprehensive description of the methodology used can serve the scientific community better than a brief description.

1. Identify putative miR–target interactions using in silico tools
  target prediction software [e.g., TargetScan (www.targetscan.org), PicTar (pictar.mdc-berlin.de), PITA (genie.weizmann.ac.il), DIANA-microT (diana.cslab.ece.ntua.gr/microT), RNA22 (cm.jefferson.edu/rna22v1.0), miRanda (www.microrna.org)],
  existing ChIP-seq databases of endogenous miR–target interactions [29, 30],
  expression profiles of miRs (www.microrna.org) and their possible targets (www.genecards.org).
2. Screen miR–target interactions using a luciferase reporter assay. To retain the full properties of the 3′UTR sequence, it is preferable to use the full-length 3′UTR.
3. Clarify the direct interaction by performing mutation analyses of the predicted miR-binding site within the 3′UTR in the context of both exogenous and endogenous miR expression.
4. Measure endogenous gene expression (at the mRNA and protein levels)
  after miR overexpression with pre-miRs or miR mimics in primary cultures and/or cell lines,
  after suppressing endogenous miR expression in primary cultures and/or cell lines.

## Abbreviations

AD: Alzheimer’s disease
AM: AntagomiR
ARPE-19: Human artificial retinal pigment epithelial cells
bEnd.3: Immortalized mouse brain endothelial cell line
BDNF: Brain-derived neurotrophic factor
C2C12: Mouse myoblast cell line
ChIP-seq: Chromatin immunoprecipitation combined with massively parallel DNA sequencing
H9c2: Rat cardiac myoblast cell line
HEK-293: Human embryonic kidney cell line
HeLa: Human cervical cancer cell line
HUVEC: Human umbilical vein endothelial cells
LNA: Locked nucleic acid
MCF7: Michigan Cancer Foundation-7 human breast cancer cell line
miR: MicroRNA
MRE: MicroRNA response element
Neuro2a: Mouse neuroblastoma cell line
NG108-15: Hybrid cell line from fused mouse neuroblastoma and rat glioblastoma cells
SCG7901: Human gastric cancer cell line
SH-SY5Y: Human neuroblastoma cell line
U-87 MG: Human glioblastoma cell line
UTR: Untranslated region
